# Supplementary material for: Prevalence of visual snow and relation to attentional absorption
Source: PLoS One. 2022 Nov 7;17(11):e0276971. doi: 10.1371/journal.pone.0276971 (PMC9639836; doi:10.1371/journal.pone.0276971)
Supplement: S1 Table — (DOCX) [file pone.0276971.s001.docx]

Table A. Descriptive statistics for low blood pressure, blood pressure drops, ophthalmological problems, computer screen time, and lifetime use of psychoactive substances.

|  | % |
| --- | --- |
| Ophthalmological problems (Study 1) | 53.1 |
| Ophthalmological problems (Study 2) | 57.0 |
| Low blood pressure | 24.2 |
| Blood pressure drops | 23.5 |
| Lifetime use of cannabis. | 45.4 |
| Lifetime use of psychedelics | 9.6 |
| Lifetime use of MDMA | 13.1 |
| Lifetime use of cocaine/stimulants | 12.7 |
| Lifetime use of any the psychoactive substances listed above | 45.8 |
