# Supplementary material for: Prevalence of visual snow and relation to attentional absorption
Source: PLoS One. 2022 Nov 7;17(11):e0276971. doi: 10.1371/journal.pone.0276971 (PMC9639836; doi:10.1371/journal.pone.0276971)
Supplement: S2 Table — (DOCX) [file pone.0276971.s002.docx]

Table B. Frequency of tinnitus.

|  | Study 1 | Study 2 |
| --- | --- | --- |
| *Estimated percentage of time with tinnitus*  0%  10%  20%  30%  40%  50%  60%  70%  80%  90%  100% | N = 482  41.9  18.3  9.1  7.1  3.7  5.0  3.1  2.1  2.5  3.1  4.1 | N = 277  52.0  10.5  9.0  4.3  6.1  3.2  4.0  4.0  2.9  1.1  2.9 |
