# Supplementary material for: Prevalence of visual snow and relation to attentional absorption
Source: PLoS One. 2022 Nov 7;17(11):e0276971. doi: 10.1371/journal.pone.0276971 (PMC9639836; doi:10.1371/journal.pone.0276971)
Supplement: S3 Table — (DOCX) [file pone.0276971.s003.docx]

Table C. Frequency of floaters and blue field entoptic phenomena.

|  | Study 1 |  | Study 1 |
| --- | --- | --- | --- |
| *Estimated percentage of time seeing floaters*  0%  10%  20%  30%  40%  50%  60%  70%  80%  90%  100% | N = 450  46.7  18.2  8.2  4.7  4.7  5.3  2.7  1.8  1.8  .4  5.6 | *Estimated percentage of time seeing blue field entoptic phenomenon*  0%  10%  20%  30%  40%  50%  60%  70%  80%  90%  100% | N = 456  66.4  12.1  4.4  3.9  1.3  2.4  1.1  1.3  .9  1.1  5.0 |
