# Supplementary material for: Prevalence of visual snow and relation to attentional absorption
Source: PLoS One. 2022 Nov 7;17(11):e0276971. doi: 10.1371/journal.pone.0276971 (PMC9639836; doi:10.1371/journal.pone.0276971)
Supplement: S4 Table — (DOCX) [file pone.0276971.s004.docx]

Table D. Pearson’s correlations between absorption and visual snow (dichotomously measured as never experiencing it vs. at least 10% of the time experiencing it).

|  | Study 1 | Study 2 | Study 3 |
| --- | --- | --- | --- |
|  | Visual snow^1^ | Visual snow^1^ | Visual snow^1^ |
| Absorption (total score)^2^ | .20*** | .26*** | .37*** |
| Altered states of consciousness | .26*** | .32*** | .36*** |
| Esthetic involvement in nature | .14** | .19*** | .26*** |
| Imaginative involvement | .15** | .19*** | .31*** |
| Synesthesia | .18*** | .20*** | .34*** |
| Extrasensory perception | .14** | .25*** | .34*** |

^1^ 0 = Never experiencing visual snow, 1= experiencing visual snow at least 10% of the time

^2^ Modified Tellegen Absorption Scale (MODTAS)

*** p < .001, ** p < .01
