# Supplementary material for: Prevalence of visual snow and relation to attentional absorption
Source: PLoS One. 2022 Nov 7;17(11):e0276971. doi: 10.1371/journal.pone.0276971 (PMC9639836; doi:10.1371/journal.pone.0276971)
Supplement: S5 Table — (DOCX) [file pone.0276971.s005.docx]

Table E. Factor loadings of the items of the Modified Tellegen Absorption Scale (MODTAS).

|  | Total sample^1^ | Study 1 | Study 2 | Study 3 |
| --- | --- | --- | --- | --- |
| Items | Factor loadings | Factor loadings | Factor loadings | Factor loadings |
| Item 1 | .53 | .56 | .45 | .57 |
| Item 2 | .40 | .50 | .43 | .37 |
| Item 3 | .57 | .58 | .52 | .62 |
| Item 4 | .57 | .53 | .55 | .62 |
| Item 5 | .66 | .64 | .65 | .71 |
| Item 6 | .53 | .53 | .55 | .53 |
| Item 7 | .61 | .65 | .56 | .65 |
| Item 8 | .57 | .58 | .53 | .58 |
| Item 9 | .63 | .65 | .61 | .62 |
| Item 10 | .56 | .58 | .56 | .54 |
| Item 11 | .71 | .67 | .71 | .75 |
| Item 12 | .64 | .63 | .66 | .63 |
| Item 13 | .56 | .54 | .58 | .56 |
| Item 14 | .63 | .60 | .62 | .66 |
| Item 15 | .63 | .61 | .69 | .62 |
| Item 16 | .72 | .72 | .74 | .73 |
| Item 17 | .60 | .59 | .64 | .57 |
| Item 18 | .61 | .61 | .63 | .60 |
| Item 19 | .63 | .66 | .58 | .64 |
| Item 20 | .69 | .68 | .72 | .70 |
| Item 21 | .59 | .60 | .57 | .58 |
| Item 22 | .61 | .63 | .62 | .57 |
| Item 23 | .62 | .65 | .64 | .61 |
| Item 24 | .66 | .67 | .71 | .61 |
| Item 25 | .56 | .61 | .60 | .47 |
| Item 26 | .50 | .53 | .49 | .48 |
| Item 27 | .66 | .65 | .69 | .65 |
| Item 28 | .56 | .52 | .56 | .59 |
| Item 29 | .65 | .63 | .66 | .65 |
| Item 30 | .64 | .56 | .71 | .71 |
| Item 31 | .57 | .59 | .49 | .64 |
| Item 32 | .68 | .69 | .68 | .67 |
| Item 33 | .60 | .60 | .63 | .57 |
| Item 34 | .61 | .62 | .62 | .60 |

^1^ Sample obtained from aggregating data from the three studies.
