# Supplementary material for: Prevalence of visual snow and relation to attentional absorption
Source: PLoS One. 2022 Nov 7;17(11):e0276971. doi: 10.1371/journal.pone.0276971 (PMC9639836; doi:10.1371/journal.pone.0276971)
Supplement: Attachment — Submitted filename: Response to Reviewers.docx [file pone.0276971.s007.docx]

Response to Reviewers

Response to Reviewer #1

*>>>>> The manuscript reads well. The authors have considered carefully each of the points I raised and have altered the manuscript appropriately. I remain concerned that in the sentence "An untested possibility is that people experiencing visual snow may be more engrossed in perceptual and imaginary experiences, because visual snow may result from hyperexcitability of the visual cortex [4-12]." Why should hyperexcitability of the visual cortex give rise to imaginary experiences? Perhaps the authors could elaborate their explanation with a sentence or two. There is in fact a little evidence of an association between hyperexcitability and imaginary experiences, as in the papers by Braithwaite et al, which the authors already cite.*

R: We added three more references to support the claim that imagination vividness is related to visual cortex activity, and based on that we noted that if the visual cortex is hyperactive, it is likely that visual imagery is particularly vivid and attention-grabbing. To support this claim, we added more references, including the study by Braithwaite that was already cited in another part of the manuscript and another one from the same author. We added to the Introduction that “Studies show that vividness of mental imagery is related to activity of primary [21,22] and association visual cortex [22,23], suggesting that if visual cortex becomes hyperactive, imaginary experiences are likely to become more vivid, and as such more likely to grab attention, at least if attention was previously directed inwards. In accordance, pharmacologically-induced visual cortex hyperactivation through LSD correlates with increases in vividness of mental imagery, but not with other psychoactive effects [24]. Additionally, more responsiveness to the pattern glare task (an index of visual cortex hyperexcitability) was associated with history of out-of-body experiences, which can be seen as an extreme case of visual imagery vividness [25,26]. Pattern-glare task responsiveness was unrelated to history of sensed presence experiences, which are not characterized by vivid visual experiences, suggesting that the visual cortex hyperexcitability does not promote altered states of consciousness in general, but rather those characterized by strong visual imagery vividness [26]”.

Below we added that “Visual snow is associated is associated with hyperexcitability of the primary and association cortex [4-12], whose activity increases as visual imagery gets more vivid [21-24]”.

References 20 to 25 cited in these parts are the following:

21. Cui X, Jeter CB, Yang D, Montague PR, Eagleman DM. Vividness of mental imagery: individual variability can be measured objectively. Vision Res 2007; 47: 474-478.

22. Dijkstra N, Bosch SE, van Gerven MAJ. Vividness of Visual Imagery Depends on the Neural Overlap with Perception in Visual Areas. J Neurosci 2017; 37: 1367-1373.

23. Fulfold J, Milton F, Salas D, Smith A, Simler A, Winlove C, Zeman A. The neural correlates of visual imagery vividness – an fMRI study and literature review. Cortex 2018; 105: 26-40.

24. Carhart-Harris RL, Muthukumaraswamy S, Roseman L, Kaelen M, Droog W, Murphy K, Tagliazucchi E, Schenberg EE, Nest T, Orban C, Leech R, Williams LT, Williams TM, Bolstridge M, Sessa B, McGonigle J, Sereno MI, Nichols D, Hellyer PJ, Hobden P, Evans J, Singh KD, Wise RG, Curran HV, Feilding AF, Nutt DJ. Neural correlates of LSD experience revealed by multimodal neuroimaging. Proc Natl Acad Sci U S A 2016; 113: 4853-4858.

25. Braithwaite JJ, Broglia E, Bagshaw AP, Wilkins AJ. Evidence for elevated cortical hyperexcitability and its association with out-of-body experiences in the non-clinical population: New findings from a pattern-glare task. Cortex 2013; 49: 793-805.

26. Braithwaite JJ, Broglia E, Brincat O, Stapley L, Wilkins AJ, Takahashi C. (2013) Signs of increased cortical hyperexcitability selectively associated with spontaneous anomalous bodily experiences in a non-clinical population. Cogn Neuropsychiatry 2013; 18: 549-573.

Response to Reviewer #2

*>>>>> I thank the authors for their revisions. As in the original manuscript, I have no issues with the data and analysis themselves, but remain cautious about what, if anything, can be concluded from these type of surveys. I would ask therefore that these issues are put more strongly in the discussion.*

*On the point that participants may be simply inclined to agree with statements, this is mentioned rather briefly, then it is argued that it is probably not an issue due to agreement with past studies. I would like to see a more reflective statement ono the limitations of the current design as a way of providing independent evidence on this point.*

R: In the subsection “Further directions for future research”, we added that “surveys allow the collection of large samples, but have the limitation of reliance on self-report. In the future, it would be useful that survey studies are complemented by qualitative data based on interviews and by test-retest studies to assess the coherence of the responses across a short period of time”.

Meanwhile a new study came to our knowledge suggesting that the prevalence of frequent or constant visual snow is similar to the one obtained in the Study 1 of our manuscript. Because this is independent research that corroborates our findings, we now cited it. As such, we added to the Introduction that “A higher prevalence of visual snow was suggested by a study with 117 participants with history of hallucinogen consumption divided in three groups. The prevalence of those with frequent or constant visual snow varied roughly between 16% and 21% before use of hallucinogens, precluding the possibility of visual snow being related to Hallucinogen Persistent Perception Disorder (HPPD) by that time [20].”

Additionally, in the subsection “Prevalence of visual snow”, we added that “Notably, the prevalence of frequent or persistent visual snow we obtained in Study 1 is similar to that obtained in a study of users of hallucinogens before they started using this type of drugs [20], which rules out that hallucinogens were part of the etiology”.

We added the following reference:

20. Irvine A, Luke D. Apophenia, absorption and anxiety: Evidence for individual differences in positive and negative experiences of Hallucinogen Persisting Perceptual Disorder. J Psychedelic Stud 2022; 6: 88-103

*>>>>> On the comparison with more rigorous and accepted prevalence statistics, again the limitations of the current study do not really seem to be highlighted clearly enough. In the new section on page 23, this issues is acknowledge, but I am left wondering what, if anything, we can conclude from the responses on migraine. It would be very help to have a much more detailed discussion of the link to proper measures of the various phenomena and where the discrepancies arise.*

R: Even if migraine and visual snow were correlated in the expected direction, we acknowledge that the measure of migraine has limitations that may lead to overestimation of migraine cases. We deleted the analyses involving migraine and related discussion. In addition, we added more details when discussing our measures of tinnitus, entoptic phenomena, and absorption.

In the subsection “Visual snow and tinnitus”, we expanded the discussion on the possible limitations of our measure of tinnitus prevalence and how it compares with other measures employed to assess tinnitus prevalence. As such, we added that “However, there are no standardized measures for tinnitus, which makes prevalence estimates uncertain. Research on tinnitus prevalence has resorted to different types of questions across studies [49]. Although we cannot rule out that our measure of tinnitus overestimated its prevalence, it is not equally possible to rule out that other measures underestimated it, especially when it occurs briefly and/or infrequently. For example, sometimes questions focus on tinnitus (“noises in your head or ears”) for more than five minutes, which may not always happen. In contrast, our measure assesses a large spectrum of tinnitus frequencies without making reference to tinnitus duration, which includes very transient and infrequent experiences. In fact, when we do not count the participants reporting frequencies of 10% (see Figure 2 and Table B in Appendix), the prevalence is inside the reported range [49]. The proportion of respondents experiencing tinnitus more than 10% of time was 40% in Study 1 and 37% in Study 2 (see Figure 2 and Table B in Appendix), which is comparable to that of the literature [49].

Other commonly used measures ask if within the past year, respondents did ever hear a sound (“buzzing, hissing, ringing, humming, roaring, machinery noise”) originating in the ear [49]. It is possible that when tinnitus is infrequent, many people do not report it, because they do not want to identify as someone who hears noises that do not have an external origin. Because our measure allows the option of responding affirmatively to the existence of infrequent tinnitus, it may avoid such identification. Only future research comparing different types of measures could clarify these issues”.

In the subsection “Visual snow and entoptic phenomena”, we expanded the discussion on the possible limitations regarding our measure of entoptic phenomena and how it compares with other measures. As such, we added that “Given the lack of standardized measures to assess entoptic phenomena, we must be cautious regarding the obtained prevalence (see Table C in Appendix). However, we believe that our measure has advantages over others, as we used graphical simulations and resorted to a scale that encompasses a large spectrum of frequency of the experiences (including only occasional experience). These are likely an improvement over measures that only rely on verbal descriptions and less nuanced scales of frequency of occurrence of entoptic phenomena. Only future research using different measures, including in-person interviews might clarify the question of the real prevalence”.

In the subsection “Visual snow and absorption”, we highlighted that the measure of absorption we used is commonly used in surveys, and it yields convergent findings with studies in other contexts. In this regard, we added that “The MODTAS is a commonly used measure of absorption with results confirming its criterion validity [37,54,61-68], including in experimental [62,65,66], and longitudinal studies [63]”.

References cited in this new part (including newly added) are the following:

37. Jamieson GA. The Modified Tellegen Absorption Scale: a clearer window on the structure and meaning of absorption. J Australian Clin Exp Hypn 2005; 33:119-139.

54. Ellero J, Costa RM. Altered states of consciousness, absorption, and sexual responsiveness. Psicol Saúde Doenças 2020; 21: 782-795.

61. Leppanen, M. L., & Kim, K. Absorption and mindfulness reflect distinct patterns of attentional control and self-related processing. J Indiv Diff 2022; 43: 143-151.

62. Timmermann C, Roseman L, Williams L, Erritzoe D, Martial C, Cassol H, Laureys S, Nutt, Carhart-Harris R. DMT models the near-death experience. Front Psychol 2018; 9: 1424.

63. Haijen ECHM, Kaelen M, Roseman L, Timmermann C, Kettner H, Russ S, Nutt D, Daws RE, Hampshire ADG, Lorenz R, Carhart-Harris RL. Predicting Responses to Psychedelics: A Prospective Study. Front Pharmacol 2018; 9:897.

64. Cardeña E, Terhune DB. Hypnotizability, personality traits, and the propensity to experience alterations of consciousness. Psychol Conscious: Theory Res Pract 2014; 1:292-307.

65. Terhune DB, Luke DP, Kaelen M, Bolstridge M, Feilding A, Nutt D, Carhart-Harris R, Ward J. A placebo-controlled investigation of synaesthesia-like experiences under LSD. Neuropsychologia 2016; 88: 28-34.

66. Studerus E, Gamma A, Kometer M, Vollenweider FX. Prediction of psylocibin response in healthy volunteers. PLoS One 2012; 7: e30800.

67. Levine A, Luke D. Apophenia, absorption and anxiety: Evidence for individual differences in positive and negative experiences of Hallucinogen Persisting Perception Disorder. J Psychedelic Stud 2022; 6: 88-103.

68. Wendler E, Schubert E. Synaesthesia, creativity and obsessive-compulsive disorder: Is there a link? Creat Res J 2019; 31: 329-334.

*>>>>> On this point, and apologies for introducing another (albeit related) point, to what extent is it possible to differentiate different types of visual snow as entirely separate phenomena based on underlying mechanisms. Based purely on personal experience, I fully recognise the experience of visual snow resulting from sudden changes in blood pressure, but would not relate this to the experience of clinical visual snow, related to cortical excitability. A concern here is that the definition is very broad, allowing for a lot of positive responses.*

R: That is an interesting point. In the subsection “Triggers of visual snow”, we added that

“Since visual snow has various triggers, it is unclear if it has always the same underlying neural mechanisms, and if these are the same occurring in untriggered visual snow; for example, it is not possible to know if visual cortex hyperexcitability is always present. One way to get a clearer picture may be to apply measures of neural activity like EEG or fMRI and compare the phenomena and their neural correlates under different induction conditions, if this is possible in the lab. This may be combined with the development of a specific questionnaire about visual snow that contains a particular focus on the comparison of different inducing conditions”.

*>>>>> Finally on the point of data availability - although a link has been I could not see the data, I think they have not been uploaded yet.*

R: Yes, we have the data ready to upload, but we have not done it yet. We will upload the data after acceptance of the manuscript.
